# An Index of Surgery

**Published:** 1884-06

**Authors:** 


*An Index of Surgery.* By C. B. Keetley, F.R.C.S. Pp. 494. Second Edition. London: Smith, Elder and Co. 1884.

We expect a book of this sort, designed for the senior student who is desirous of passing an examination, to be a heterogeneous hash of disjecta membra lopped off the various monuments of surgical literature, and served up crude, incoherent, and unelaborated under the author's labels as genuine intellectual pabulum. But we find something very different in this case. Here we have honest work of a high order—independent, laborious, and trustworthy. And it is more than an index, it is a dictionary, and a by no means incomplete one. The language is much abbreviated in most places; but here and there, especially where some subject peculiarly his own is treated, the author expands into flowing and pithy sentences, which almost make us wish that the whole work had been so written.

The work is singularly equal throughout. There is very little to be found fault with, and much to be commended. Among the former we would instance the mode of administering ether which he recommends; viz., on a towel folded conically, with a sponge at the bottom. Surely fracture of the neck of the humerus is not the only or even the chief surgical lesion which is liable to be confounded with dislocation of the shoulder-joint, and fatty embolism is not confined to cases of *compound* fracture. On the other side, the most striking excellence of the work is, in our opinion, the surprising manner in which it is kept up to date. Not only have we excellent descriptions of such subjects of recent interest as The Localization of Cerebral Injuries; Wiring of the Patella; Microscopic Organisms; Excision of the Rectum; The Modern Operative Treatment for Radical Cure of Hernia and so forth; but we find frequent references to papers bearing on many of the subjects, which must have appeared as the work was passing through the press. No better evidence of the vitality of the work could be adduced than the frequent appearance of such references to modern papers, and the liberal-minded appreciation with which the author has studied them. There is none of the affectation of the mere bibliographer about this. The author quotes a paper because he has read it, and appreciated it, and found it good; papers of another sort he ignores.

The author in this way has succeeded in building up a work which is unique of its kind. Nowhere else in the English language have we so much surgical truth presented in so small a compass, so well arranged, so thoroughly up to date, and withal so readable. We heartily commend it to the perusal of our readers.

